# Маркеры активизации стрессорной системы у пациентов с сахарным диабетом 1 типа на фоне гипогликемии

**DOI:** 10.14341/probl13318

**Published:** 2025-05-20

**Authors:** Р. А. Карамуллина, С. М. Исмаилова, Е. Д. Пешева, И. В. Полубояринова, М. Г. Полуэктов, В. В. Фадеев

**Affiliations:** Первый Московский государственный медицинский университет имени И.М. Сеченова (Сеченовский университет); Первый Московский государственный медицинский университет имени И.М. Сеченова (Сеченовский университет); Первый Московский государственный медицинский университет имени И.М. Сеченова (Сеченовский университет); Первый Московский государственный медицинский университет имени И.М. Сеченова (Сеченовский университет); Первый Московский государственный медицинский университет имени И.М. Сеченова (Сеченовский университет); Первый Московский государственный медицинский университет имени И.М. Сеченова (Сеченовский университет)

**Keywords:** сахарный диабет, гипогликемия, стресс, нарушения сна

## Abstract

**ОБОСНОВАНИЕ:**

ОБОСНОВАНИЕ. Как правило, гипогликемический эпизод развивается вследствие неадекватности введенной дозы инсулина сообразно текущей физиологической ситуации. Активизированные системы, направленные на повышение уровня глюкозы крови, являются предвестниками гипогликемии и маркерами выраженности гиперинсулиемии. По этому определение их компонентов может служить более тонким и чувствительным вариантом оценки физиологичности того или иного варианта инсулинотерапии.

**ЦЕЛЬ:**

ЦЕЛЬ. Изучить маркеры (биохимические, клинические и морфологические) и степень активации стрессорной системы в зависимости от частоты гипогликемических эпизодов на фоне проводимой инсулинотерапии у пациентов с сахарным диабетом 1 типа (СД1).

**МАТЕРИАЛЫ И МЕТОДЫ:**

МАТЕРИАЛЫ И МЕТОДЫ. Проведено наблюдательное одномоментное клиническое исследование с участием 74 па циентов с СД1. Все пациенты прошли обследование, включавшее оценку анамнеза гипогликемических состояний, качества жизни по данным опросника SF-36, уровней адренокортикотропного гормона (АКТГ), инсулиноподобного фактора роста-1 (ИФР-1), кортизола, С-реактивного белка (СРБ), определение коагулограммы, суточной экскреции кортизола с мочой. Оценка особенностей сна пациентов проводилась на основании результатов заполнения опро сников: анкеты сна; Эпвортской шкалы сонливости. Пациентам проводилась ночная полисомнография (ПСГ) с рас шифровкой по стандартам AASM 2012.

**РЕЗУЛЬТАТЫ:**

РЕЗУЛЬТАТЫ. У пациентов с более высокой частотой эпизодов гипогликемий на всех этапах зафиксировано снижение уровня ИФР-1 (140 [123:162]; 98 [93:121], р=<0,005), хуже показатели качества жизни по всем шкалам опросника SF-36 (95 [88:100]; 84 [77:92], 0,001). По мере увеличения частоты эпизодов гипогликемий, по данным ПСГ, отмечается увеличение количества пробуждений более 3 минут (2 [1:3]; 3 [2:4]; р=0,03), увеличение продолжительностей времени в кровати (ВВК) (493,1 [463,95:513,4]; 536,2 [511,6:551]; р=0,03), времени периода сна (437,5 [430,05:468]; 489 [471,5:519], р=0,006), общего времени сна (382,5 [321,75:422]; 439 [409,5:486], р=0,008).

**ЗАКЛЮЧЕНИЕ:**

ЗАКЛЮЧЕНИЕ. Развитие эпизода гипогликемии, как правило, сопровождается активацией стрессорной системы, однако повторные эпизоды гипогликемий приводят к истощению стрессорной системы, о чем свидетельствует снижение уровня ИФР-1 у пациентов с частыми эпизодами гипогликемий. Эпизоды гипогликемий, возникающие не только в ночное время, приводят к нарушению структуры сна в виде увеличения частоты ночных пробуждений.

## Abstract (en)

**BACKGROUND:**

BACKGROUND: Usually, a hypoglycemic episode occurs due to inadequacy of the administered insulin dose in accordance with the current physiological situation. Activated systems aimed at increasing blood glucose levels serve as precursors of hypoglycemia and markers of the severity of hyperinsulinemia. Therefore, determining their components can serve as a more subtle and sensitive approach to assessing the physiological appropriateness of different insulin therapy options.

**AIM:**

AIM: To investigate the markers (biochemical, clinical, and morphological) and the degree of activation of the stress system preceding the development of hypoglycemic episodes in patients with type 1 diabetes (T1D) undergoing insulin therapy.

**MATERIALS AND METHODS:**

MATERIALS AND METHODS: A cross-sectional observational clinical study was conducted involving 74 patients with type 1 diabetes (T1D). All patients underwent examination, which included assessment of the history of hypoglycemic episodes, quality of life using the SF-36 questionnaire, levels of adrenocorticotropic hormone (ACTH), insulin-like growth factor-1 (IGF-1), cortisol, C-reactive protein (CRP), coagulation profile, and 24-hour urinary cortisol excretion. Evaluation of patients’ sleep characteristics was performed based on the results of completed questionnaires: Sleep Questionnaire and Epworth Sleepiness Scale. Patients underwent overnight polysomnography (PSG) with interpretation according to the AASM 2012 standards.

**RESULTS:**

RESULTS: Patients with a higher frequency of hypoglycemic episodes showed a decrease in IGF-1 levels at all stages (140 [123:162]; 98 [93:121], p=0.005), worse quality of life scores across all domains of the SF-36 questionnaire (95 [88:100]; 84 [77:92], p=0.001). As the frequency of hypoglycemic episodes increased, polysomnography data revealed an increase in the number of awakenings lasting more than 3 minutes (2 [1:3]; 3 [2:4]; p=0.03), increased time spent in bed (493.1 [463.95:513.4]; 536.2 [511.6:551]; p=0.03), increased sleep duration (437.5 [430.05:468]; 489 [471.5:519], p=0.006), and in creased total sleep time (382.5 [321.75:422]; 439 [409.5:486], p=0.008).

**CONCLUSION:**

CONCLUSION: An increase in the frequency of hypoglycemic episodes should be accompanied by activation of the stress response system; however, repeated episodes of hypoglycemia lead to depletion of the stress response system, as evidenced by a decrease in the level of IGF-1 in patients with frequent hypoglycemic episodes. Hypoglycemic episodes occurring not only during night time but also at other times disrupt the sleep structure by increasing the frequency of nocturnal awaken ings.

## ОБОСНОВАНИЕ

Механизм контррегуляторного ответа на снижение уровня глюкозы крови был описан в середине 1970-х — начале 1980-х годов в ходе исследований с непрерывной инфузией инсулина для индукции гипогликемии [1][2]. Было показано, что, помимо снижения уровня циркулирующего инсулина, существуют другие механизмы, предотвращающие развитие гипогликемии [2]. Впоследствии был проведен ряд крупных исследований, посвященных их выявлению и оценке роли каждого из них в реализации контринсулярного ответа.

В физиологических условиях, без нарушенного контррегуляторного ответа, снижение уровня глюкозы плазмы запускает каскад защитных механизмов: 1) снижение секреции инсулина бета-клетками поджелудочной железы; 2) увеличение секреции глюкагона; 3) стимуляция секреции катехоламинов, гормона роста и кортизола [3]. Кроме того, появляются вегетативные и нейрогликопенические симптомы, побуждающие человека к купированию данного состояния [4].

При этом у пациентов с сахарным диабетом 1 типа (СД1) и у пациентов с большим стажем сахарного диабета 2 типа (СД2) может быть нарушен защитный ответ на гипогликемию [5]. Среди основных дефектов механизма контррегуляторного ответа можно выделить:

Любой фактор, воздействующий на организм, в силу особенностей своей природы вызывает комплекс адаптивных изменений сообразно силе и качеству раздражения. Так, например, изменения в системе гемостаза, возникающие для немедленной адаптации на воздействие раздражителя, включают комплекс стереотипных реакций, обеспечивающих готовность системы к остановке кровотечения. Вне зависимости от природы стрессора происходит нарастание коагуляции с подавлением фибринолитической, антикоагулянтной систем [15]. Подобно этому стресс-системы, препятствующие снижению уровня гликемии и развитию гипогликемии, приводят к изменению функциональной активности различных систем организма, в том числе эндокринной, иммунной [8][9] и сердечно-сосудистой систем [10][11][12][13][14].

Механизм контррегуляторного ответа и стадии секреции гормонов стрессорной системы в ответ на свершившийся эпизод гипогликемии давно известны. Однако клиническое значение подобной реакции изучено недостаточно. В связи с этим изучение маркеров стрессорной системы позволит проанализировать возможность и значимость прямой оценки активности контррегуляторной системы как дополнительного инструмента определения компенсации углеводного обмена и качества проводимой сахароснижающей терапии.

## ЦЕЛЬ ИССЛЕДОВАНИЯ

Изучить маркеры (биохимические, клинические и морфологические) и степень активации стрессорной системы в зависимости от частоты гипогликемических эпизодов на фоне проводимой инсулинотерапии у пациентов с СД1.

## МАТЕРИАЛЫ И МЕТОДЫ

## Место и время проведения исследования

Место проведения. Клиника эндокринологии Первого МГМУ им. И.М. Сеченова г. Москвы.

Время исследования. Исследование проводилось с октября 2020-го по сентябрь 2022 гг.

## Изучаемая популяция

В исследование включено 74 пациента с СД1 (43 мужчины и 31 женщина). На момент включения средний возраст участников составил 30 лет [24–37] при стаже заболевания 14 лет [8–22]. Общая характеристика включенных в исследование представлена в таблице 1.

**Table:** Таблица 1. Общая характеристика пациентов, включенных в исследование СДИ — суточная доза инсулина; МИИ — множественные инъекции инсулина; НПВИ — непрерывное подкожное введение инсулина.

Показатель	Все участники
Соотношение мужчин/женщин, абс. (%)	43/31 (58/42)
Возраст, лет	30 [ 24; 37]
Стаж СД1, лет	14 [ 8; 22]
СДИ, Ед	38,5 [ 30; 51]
ИМТ, кг/м²	23 [ 21; 25]
HbA1c, %	7,6 [ 6,8; 8,8]
Соотношение МИИ/ НПВИ, абс. (%)	39/35 (52/48)

Критериями включения были: возраст пациентов более 18 лет; подтвержденный СД1, диагностированный не менее, чем за 1 год до включения в исследование.

Критериями исключения были: постменопауза, беременность, прием седативных и других психотропных препаратов, депрессия и другие психические расстройства, наличие поздних осложнений СД (автономная нейропатия, ХБП С3–5), наличие заболеваний гепатобилиарной системы, прием эстрогенсодержащих препаратов.

Все 74 пациента, включенные в исследование, были последовательно трижды разделены на 2 группы в зависимости от частоты эпизодов гипогликемий (рис. 1). При первом разделении все пациенты были распределены на 2 группы (1а; 1в): пациенты группы 1а (n=15) — пациенты с частотой эпизодов гипогликемий 0–1 эпизод в неделю, пациенты группы 1в (n=59) — пациенты с частотой эпизодов гипогликемий >1 эпизода в неделю. При втором разделении к группе пациентов (1а) с частотой эпизодов гипогликемий 0–1 эпизод в неделю из предыдущего разделения были добавлены пациенты с частотой эпизодов гипогликемий 1–3 эпизода в неделю, совместно сформировав группу 2а «пациенты с частотой эпизодов гипогликемий ≤3 эпизодов в неделю» (n=34). Вторую группу 2в при 2-м разделении составили пациенты с частотой эпизодов гипогликемий >3 эпизодов в неделю, соответственно (n=40). При 3-м (последнем) разделении к группе пациентов 2а с частотой эпизодов гипогликемий ≤3 эпизодов в неделю были добавлены пациенты с частотой эпизодов гипогликемий 4–5 в неделю, составив, соответственно, группу «пациенты с частотой эпизодов гипогликемий ≤5 эпизодов в неделю» 3а (n=49). Вторую группу 3в при 3-м разделении составили пациенты с частотой эпизодов гипогликемий >5 эпизодов в неделю (n=25).

**Figure:**
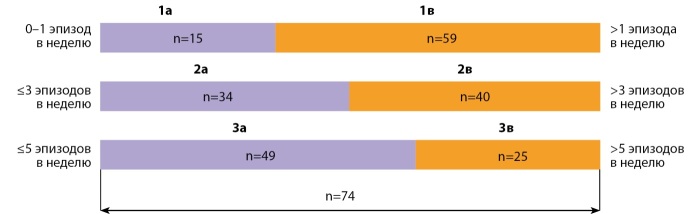
Рисунок 1. Распределение пациентов по группам.

Сравнительная оценка клинико-анамнестических показателей представлена в таблице 2:

1 группа (n=15) — пациенты с частотой эпизодов гипогликемий 0–1 эпизодов в неделю;

2 группа (n=59) — пациенты с частотой эпизодов гипогликемий >1 эпизода в неделю.

**Table:** Таблица 2. Клинико-анамнестическая характеристика пациентов в анализируемых группах Примечание. * — р<0,05.Ме [ 25:75] — медиана [ 25-й процентиль: 75-й процентиль].

Показатель, Ме [ 25:75]	Группа 1а (n= 5)	Группа 1в (n=59)	Р
Стаж СД1, лет	10 [ 6:17]	15 [ 11:23]	0,041*
Возраст, лет	31 [ 24:35]	30 [ 24:37]	0,741
ИМТ, кг/м²	21,7 [ 21:24]	23,4 [ 22:25]	0,154
HbA1c, %	9,5 [ 7,4:9,95]	7,6 [ 6,75:8,3]	0,017*
Соотношение МИИ/ НПВИ, абс. (%)	10/5 (69/31)	30/29 (52/48)	0,276
СДИ, Ед	47 [ 38:62]	36 [ 30:48]	0,034*
СДИ, Ед/кг	0,74 [ 0,59:0,88]	0,55 [ 0,45:0,7]	0,036*

Сравнительная оценка клинико-анамнестических показателей при следующем разделении представлена в таблице 3:

1 группа (n=34) — пациенты с частотой эпизодов гипогликемий ≤3 эпизодов в неделю;

2 группа (n=40) — пациенты с частотой эпизодов гипогликемий >3 эпизодов в неделю.

**Table:** Таблица 3. Клинико-анамнестическая характеристика пациентов в анализируемых группах Примечание. * — р<0,05.

Показатель, Ме [ 25:75]	Группа 2а (n=34)	Группа 2в (n=40)	Р
Стаж СД1, лет	10,5 [ 8:17]	17 [ 12,5:23,5]	0,01*
Возраст, лет	31 [ 24:38]	30 [ 24:36]	0,892
ИМТ, кг/м²	22,88 [ 21,65:25,2]	23,1 [ 21:24,7]	0,905
HbA1c, %	7,85 [ 6,9:9,5]	7,6 [ 6,8:8,2]	0,348
Соотношение МИИ/ НПВИ, абс. (%)	24/10 (71/29)	16/24 (40/60)	0,009*
СДИ, Ед/кг	0,66 [ 0,5:0,85]	0,5 [ 0,45:0,7]	0,075
СДИ, ЕД	44 [ 36:61]	35 [ 29:45]	0,005*

Сравнительная оценка клинико-анамнестических показателей при 3-м разделении представлена в таблице 4:

1 группа (n=49) — пациенты с частотой эпизодов гипогликемий ≤5 эпизодов в неделю;

2 группа (n=25) — пациенты с частотой эпизодов гипогликемий >5 эпизодов в неделю.

**Table:** Таблица 4. Характеристика участников: 1 группа — пациенты с частотой эпизодов гипогликемий ≤5 эпизодов в неделю; 2 группа — пациенты с частотой эпизодов гипогликемий >5 эпизода в неделю Примечание. * — р<0,05.

Параметр Ме [ 25:75]	Группа 3а (n=49)	Группа 3в (n=25)	Р
Стаж	13 [ 8:18]	17 [ 13:27]	0,012*
Возраст	30 [ 24:37]	30 [ 24:36]	0,814
ИМТ, кг/м²	23 [ 22:25]	22 [ 21:25]	0,315
HbA1c, %	7,7 [ 6,9:9,5]	7,6 [ 6,8:8]	0,213
Соотношение МИИ/ НПВИ, абс. (%)	31/18 (63/37)	9/16 (36/64)	0,027*
СДИ, Ед	40 [ 32:53]	34 [ 28:42]	0,047*
СДИ	0,6 [ 0,5:0,8]	0,5 [ 0,47:0,7]	0,359

## Дизайн исследования

Проведено одноцентровое наблюдательное одномоментное клиническое исследование.

## Методы

Все пациенты прошли обследование, включавшее сбор анамнеза, физикальное исследование с оценкой антропометрических данных и измерением артериального давления (АД), оценку показателей углеводного обмена (HbA1c, гликемический профиль), оценку анамнеза гипогликемических состояний, наличия и выраженности поздних осложнений СД. Также были проведены: оценка качества жизни по данным опросника SF-36, уровней секреции адренокортикотропного гормона (АКТГ), инсулиноподобного фактора роста-1 (ИФР-1), кортизола, С-реактивного белка (СРБ), определение коагулограммы, суточной экскреции кортизола с мочой. Оценка особенностей сна пациентов проводилась на основании результатов заполнения опросников: анкеты сна; Эпвортской шкалы сонливости. Пациентам проводилась ночная полисомнография с расшифровкой по стандартам AASM 2012.

Для оценки лабораторных показателей проводился забор крови из локтевой вены натощак в 08:00. Оценивались следующие лабораторные показатели:

1) гликированный гемоглобин оценивался методом жидкостной ионообменной хроматографии высокого давления на анализаторе D-10, наборы «Bio-Rad», США (4,8–6,0%);

2) гормональные исследования крови (ИФР-1, АКТГ, кортизол крови) проводились стандартными наборами на иммунохемилюминесцентном анализаторе Immulite 2000 (Siemens, США); ИФР-1 (88–537 нг/мл), АКТГ (0–10,2 пмоль/л), кортизол (119–618 нмоль/л). Определение свободного кортизола в суточной моче проводилось иммунохемилюминесцентным методом на аппарате Beckman Coulter AU 5820 (США) с предварительной экстракцией диэтиловымэфиром (1,5–63 мкг/сут);

3) исследование биохимических параметров крови проводилось при помощи автоматического биохимического анализатора Beckman Coulter AU 5820 (США) по стандартным методикам с использованием соответствующих реактивов; СРБ (0–5 мг/л);

4) коагулологические исследования проводились при помощи автоматического коагулометра ACL TOP 750: фибриноген (1,8–4 г/л), ПТВ (9,4–12,5 сек), ТВ (15,8–24,9 сек), МНО (0,9–1,16), АЧТВ (0,75–1,25), антитромбин 3 (80–120 %).

## Методика оценки качества жизни «SF-36»

Опросник включает в себя 36 вопросов о физических, физиологических и социальных сферах жизни. Вопросы формируют 8 шкал качества жизни (КЖ). Различные шкалы включают от 2 до 10 пунктов. Каждый пункт используется только одной определенной шкалой. В соответствии со стандартной процедурой обработки значение каждой шкалы выражается в нормированных баллах и колеблется в диапазоне от 0 до 100, где 0 — наихудшее, а 100 — наилучшее качество жизни.

## Исследование нарушений сна

Оценка особенностей сна пациентов проводилась на основании результатов заполнения опросников: анкеты сна; Эпвортской шкалы сонливости. Также пациентам проводилась полисомнография. В ходе полисомнографии оценивались такие показатели, как: 6 каналов ЭЭГ в монополярных отведениях Fp1A2, Fp2A1, C3A2, C4A1, O1A2, O2A1, 2 канала ЭОГ, 1 канал подбородочной ЭМГ, 1 канал ЭКГ, регистрация показателей дыхания во сне с записью ороназального потока воздуха, дыхательных движений грудной и брюшной стенок, шума дыхания, уровня насыщения крови кислородом (сатурации) и положения тела в постели с параллельным видеомониторированием (без адаптационной ночи). Также оценивалась структура сна: время в кровати, время периода сна (ВПС), общее время сна (ОВС), бодрствование во время сна, эффективность сна, количество пробуждений, время засыпания, фаза быстрого сна (ФБС), латентность ФБС, продолжительность и латентность стадий сна; показатели расстройства дыхания во сне и движения конечностями.

## Оценка частоты гипогликемий

Оценка частоты гипогликемий (снижение гликемии <3,9 ммоль/л) проводилась по результатам опроса пациентов, уточнялось наличие эпизодов тяжелой гипогликемии (эпизоды, сопровождавшиеся потерей сознания или требующие помощи других лиц). Ведение дневника самоконтроля было рекомендовано в течение 10–14 дней перед включением в исследование.

## Статистический анализ

Статистический анализ проведен при помощи пакета статистических программ IBM SPSS Statistics v.23. Данные представлены в виде медианы и интерквартильного размаха — Ме (25; 75), где Ме — медиана, 25 — первый квартиль, 75 — третий квартиль. Для оценки значимости различий данных в группах применялись метод Манна-Уитни (для двух независимых групп). Сравнение двух групп по количественному показателю выполнялось с помощью U-критерия Манна-Уитни. Критический уровень значимости при проверке статистических гипотез принимался равным 0,05.

## Этическая экспертиза

Исследование было одобрено на заседании Локального этического комитета ФГАОУ ВО «Первый Московский государственный медицинский университет имени И.М. Сеченова (Сеченовский Университет)» Минздрава России, протокол №01-21 от 22.01.2021.

## РЕЗУЛЬТАТЫ

В первой части исследования пациенты были разделены на 2 группы: группа 1а — пациенты с частотой эпизодов гипогликемий ≤1 в неделю, группа 1в — пациенты с частотой эпизодов гипогликемий >1 в неделю.

В отличие от пациентов с частотой гипогликемий ≤1 в неделю, у пациентов с частотой эпизодов гипогликемий >1 в неделю выявлено статистически значимое снижение уровней ИФР-1, антитромбина-3. По результатам ПСГ, в 1 группе выявлено статистически значимое уменьшение количества пробуждений продолжительностью более 3 минут, увеличение продолжительности (мин) и представленности (%) стадии 2 сна, выше индекс десатурации (ИД) по сравнению с группой 2. При этом у пациентов в группе 2 зафиксировано увеличение представленности стадии 3 и продолжительности фазы быстрого сна (ФБС) по сравнению с группой 1. Также в группе 2 (частота гипогликемий более 1 в неделю) был ниже общий балл по результатам опросника SF-36 (рис. 2).

**Figure:**
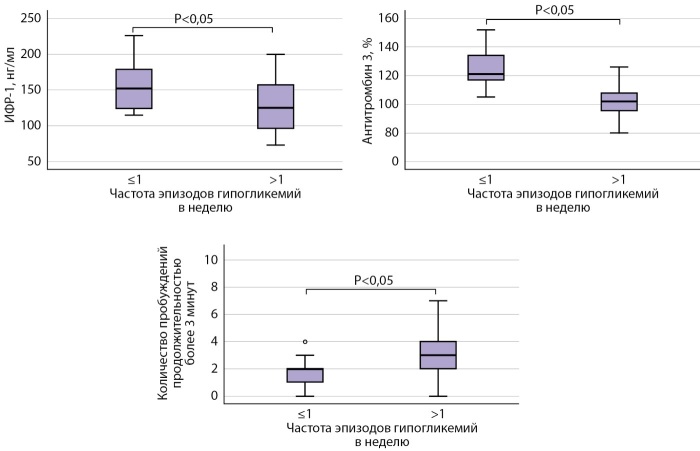
Рисунок 2. Сравнительная оценка уровней ИФР-1 и антитромбина-3, а также количества пробуждений продолжительностью более 3 минут у пациентов с частотой эпизодов гипогликемий ≤1 в неделю и >1 в неделю.

Различия остальных исследуемых параметров оказались статистически незначимыми.

Во второй части работы пациенты были разделены на следующие 2 группы: 2а группу составили пациенты с частотой эпизодов гипогликемий ≤3 в неделю, 2в группу — пациенты с частотой эпизодов гипогликемий >3 в неделю. У пациентов с частыми эпизодами гипогликемии (>3 эпизодов гипогликемии в неделю) выявлены статистически значимые более низкие уровни ИФР-1, антитромбина-3, СРБ по сравнению с пациентами с частотой эпизодов гипогликемий ≤3 в неделю. При анализе таких показателей, как АКТГ, общий кортизол, ПТВ, ТВ, фибриноген, МНО, АЧТВ, статистически значимых различий между группами не получено. Также не было зафиксировано статистически значимых различий и при оценке суточной экскреции кортизола с мочой. По данным ПСГ, в группе 2а выявлено меньшее количество пробуждений продолжительностью более 3 мин по сравнению с пациентами группы 2в. Также, по результатам опросника SF-36, у пациентов группы 2а выше как общий балл, так и балл психического компонента по сравнению с участниками группы 2в (рис. 3).

**Figure:**
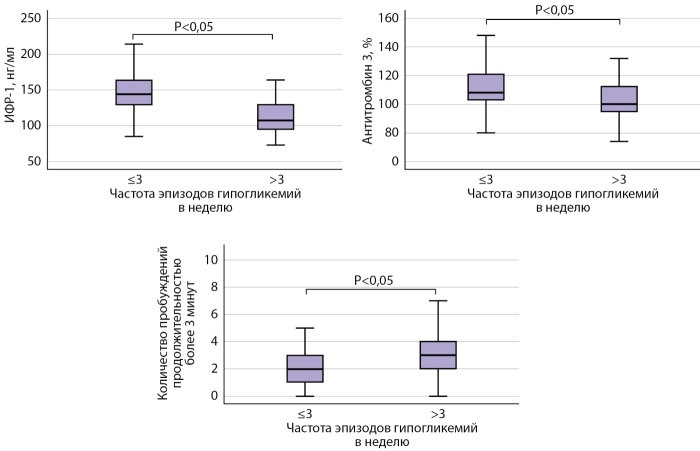
Рисунок 3. Сравнительная оценка уровней ИФР-1, антитромбина 3 и количества пробуждений продолжительностью более 3 минут у пациентов с частотой эпизодов гипогликемий ≤3 в неделю и >3 в неделю.

При сравнении пациентов с частотой гипогликемий ≤5 эпизодов в неделю (группа 3а) с пациентами с частотой эпизодов гипогликемий >5 эпизодов в неделю (группа 3в) выявлено статистически значимое снижение уровня ИФР-1 в группе 3в. По данным ПСГ, в группе 3в статистически значимое увеличение продолжительности времени в кровати (ВВК), время периода сна (ВПС), общего времени сна (ОВС). Также в группе пациентов с более высокой частотой гипогликемий по результатам опросника SF-36 выявлены более низкие баллы физического и психического компонентов здоровья и более низкий общий балл (рис. 4).

**Figure:**
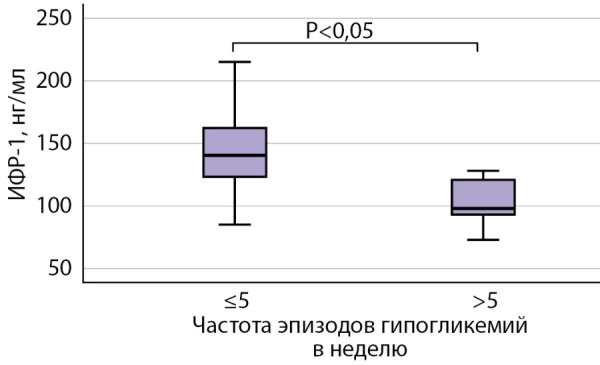
Рисунок 4. Сравнительная оценка уровня ИФР-1 у пациентов с частотой эпизодов гипогликемий ≤5 в неделю и >5 в неделю.

На рисунке 5 представлены параметры, определявшие статистически значимые различия между группами пациентов с различной частотой эпизодов гипогликемий.

**Figure:**
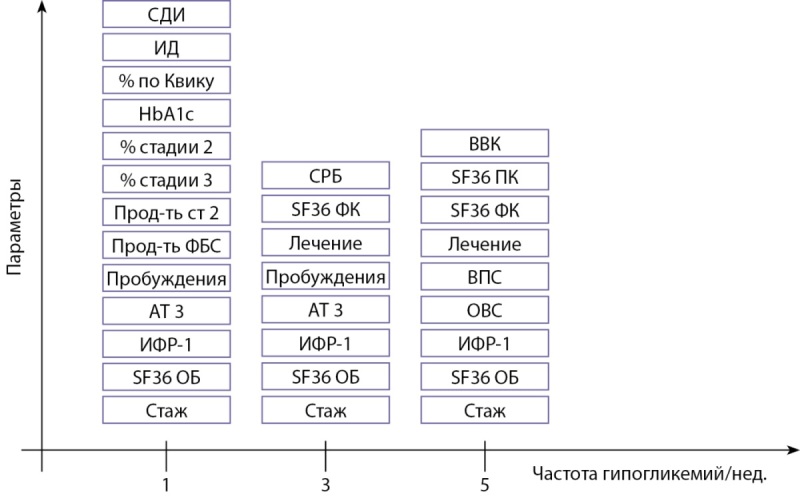
Рисунок 5. Статистически значимые различия в зависимости от частоты гипогликемий.

## ОБСУЖДЕНИЕ

В результате проведенного исследования выявлено, что у пациентов с более высокой частотой эпизодов гипогликемий зафиксировано снижение уровня ИФР-1. Также анализ стандартизированных показателей качества жизни показал, что группа пациентов с более редкими эпизодами гипогликемий ожидаемо имела лучшие показатели качества жизни по всем шкалам опросника SF-36 по сравнению с группой пациентов с частыми эпизодами гипогликемий. При анализе анамнестических данных обращало на себя внимание, что пациенты с более высокой частотой гипогликемических эпизодов чаще находились на инсулинотерапии с применением технологии непрерывного подкожного введения инсулина, а также имели более продолжительный стаж СД1. Кроме того, по мере увеличения частоты эпизодов гипогликемий, по данным ПСГ, отмечается ряд изменений характеристик сна, таких как увеличение количества пробуждений более 3 минут, увеличение продолжительности времени в кровати (ВВК), времени периода сна (ВПС), общего времени сна (ОВС).

Как было отмечено ранее, гипогликемия приводит к ряду реакций, координируемых ЦНС, — гормональным, нейрофизиологическим и когнитивным [16][17]. Пороговые значения для их развития были экспериментально определены у людей без нарушения углеводного обмена [18]. У пациентов с СД1 гликемические пороги для активации контррегуляторной системы динамичны и изменяются под влиянием ряда факторов, включая предшествующие эпизоды гипогликемии [6][19][20]. Повторные эпизоды гипогликемий приводят к ослаблению контррегуляторного ответа и снижению порога активации компенсаторных реакций [20][21]. И поскольку ГР является частью контррегуляторной системы, более низкие значения уровня ИФР-1 у пациентов с более частыми эпизодами гипогликемии могут быть следствием рецидивирующих гипогликемий. Аналогичные результаты были продемонстрированы в исследовании Nielsen L. и соавт. Так, у беременных пациенток с СД1 с частыми тяжелыми эпизодами гипогликемии уровень ИФР-1 был ниже по сравнению с пациентками, у которых не было зафиксировано гипогликемий [22]. Однако результаты данного исследования не согласуются с результатами исследования Juul A., в котором не было выявлено статистически значимой связи между частотой эпизодов гипогликемий и уровнем ИФР-1 [23].

Необходимо отметить тот факт, что в настоящее время в лечении пациентов с СД1 важное место занимают вопросы, связанные с улучшением качества жизни пациентов с СД, вовлечением их в полноценную активную трудовую деятельность, поэтому особый интерес стали представлять проблемы нарушения сна как неотъемлемой части существования человека, занимающей более трети времени его жизни [24]. Установлено, что, с одной стороны, активизированные стрессорные системы приводят к развитию нарушений сна, с другой — преобладание симпатического отдела вегетативной нервной системы (ВНС) над парасимпатической, повышение уровня кортизола в результате расстройства сна потенциально могут оказывать негативное влияние на гликемический контроль у пациентов с СД1, способствуя формированию патогенетического «порочного круга». Кроме того, нарушения сна в свою очередь приводят к развитию сниженной работоспособности, утомляемости, нарушению деятельности сердечно-сосудистой, центральной нервной систем, развитию синдрома выгорания и потере гликемического контроля.

В нашем исследовании у пациентов с более высокой частотой гипогликемий были выявлены более частые эпизоды пробуждения продолжительностью более 3 минут. Данные изменения могут быть следствием как развития непосредственно симптомов гипогликемии (потоотделение, дрожь, сердцебиение) в ночное время, которые нарушают целостность сна, так и следствием соматической и корковой гиперактивации. Соматическая гиперактивация, развивающаяся на фоне частых гипогликемий, является проявлением повышенной готовности к стрессу и сниженного порога восприятия внешних и внутренних раздражителей во время сна в результате преобладания тонуса симпатической нервной системы и дисбаланса тормозных и активирующих систем головного мозга. При этом при разделении пациентов на группы 3а и 3в не было выявлено статистически значимых различий по количеству пробуждений. В исследованиях B. Schultes и соавт. [25] и P.E. Cryer и соавт. [26] было установлено, что при снижении уровня гликемии во время ночного сна пациенты с СД1 по сравнению с участниками без нарушений углеводного обмена пробуждались реже. Авторы исследования связывают полученные результаты с развитием ослабления контррегуляторного ответа и снижением порога активации компенсаторных реакций в результате рецидивирующих эпизодов гипогликемий у данной группы пациентов. Вероятно, в проведенном нами исследовании на третьем этапе отсутствие увеличения пробуждений более 3 минут обусловлено подавлением симпатоадреналовых реакций, что согласуется с результатами ранее проведенных исследований.

Интенсифицированный режим инсулинотерапии направлен на достижение целевых показателей гликемии при сохранении высокого качества жизни пациента. Однако подобный более жесткий гликемический контроль сопряжен с увеличением частоты развития такого осложнения инсулинотерапии, как гипогликемия [27].

К настоящему моменту проведено большое количество клинических исследований, посвященных сравнению различных аспектов (в т.ч. эффективность, влияние на развитие поздних осложнений СД) применения систем непрерывного подкожного введения инсулина (НПВИ) и интенсифицированной инсулинотерапии в режиме множественных инъекций (МИИ). В большинстве проведенных исследований было выявлено статистически значимое снижение уровня гликированного гемоглобина на фоне инсулинотерапии в режиме НПВИ по сравнению с инсулинотерапией в режиме МИИ [28], как при применении аналогов инсулина ультракороткого действия, так и инсулина короткого действия [29] (HbA1c: -0,29 [ -0,46; -0,13] против -1,93 [ -1,84; -0,42]%). В ряде исследований сообщалось о снижении частоты эпизодов тяжелой гипогликемий на фоне НПВИ. При этом статистически значимой разницы между частотой эпизодов легкой гипогликемий при сравнении инсулинотерапии в режимах НПВИ и МИИ получено не было [26][30]. В нашем исследовании у пациентов, находящихся на инсулинотерапии в режиме НПВИ, частота эпизодов гипогликемий была статистически значимо выше.

## Ограничения исследования

К ограничениям нашего исследования можно отнести небольшой размер выборки, а также тот факт, что в исследовании сведения о частоте гипогликемий были получены путем опроса пациента, не проводилось непрерывного мониторирования глюкозы для объективизации данных.

## ЗАКЛЮЧЕНИЕ

Выявленные в ходе исследования маркеры стрессорной системы могут быть в дальнейшем использованы в качестве дополнительного инструмента оценки физиологичности различных вариантов инсулинотерапии в клинической практике, а также в научной оценке новых технологий лечения СД.

## ДОПОЛНИТЕЛЬНАЯ ИНФОРМАЦИЯ

Источник финансирования. Исследование и публикация статьи осуществлены на личные средства авторского коллектива.

Конфликт интересов. Авторы данной статьи подтвердили отсутствие конфликта интересов, о котором необходимо сообщить.

Благодарность. Авторы выражают искреннюю благодарность пациентам, принявшим участие в проведении исследования.

Участие авторов. Все авторы одобрили финальную версию статьи перед публикацией, выразили согласие нести ответственность за все аспекты работы, подразумевающую надлежащее изучение и решение вопросов, связанных с точностью или добросовестностью любой части работы.
